# Applying the Effective Programme Coverage framework to assess gaps in HIV prevention programmes for female sex workers and men who have sex with men in Nairobi, Kenya: findings from an expanded Polling Booth Survey

**DOI:** 10.1002/jia2.26240

**Published:** 2024-07-10

**Authors:** Parinita Bhattacharjee, Leigh McClarty, Shajy Isac, Joshua Kimani, Faran Emmanuel, Rhoda Kabuti, Antony Kinyua, Bernadette Kina Kombo, Collins Owek, Helgar Musyoki, Anthony Kiplagat, Peter Arimi, Souradet Yuh‐Nan Shaw, Monica Gandhi, Siobhan Malone, James Blanchard, Geoff Garnett, Marissa L. Becker

**Affiliations:** ^1^ Institute for Global Public Health University of Manitoba Winnipeg Manitoba Canada; ^2^ Partners for Health and Development in Africa Nairobi Kenya; ^3^ The Global Fund Geneva Switzerland; ^4^ Ministry of Health Nairobi County Kenya; ^5^ University of California San Francisco California USA; ^6^ Bill and Melinda Gates Foundation Seattle Washington USA

**Keywords:** Kenya, HIV, female sex workers, men who have sex with men, effective programme coverage, measurement methods

## Abstract

**Introduction:**

Measuring the coverage of HIV prevention services for key populations (KPs) has consistently been a challenge for national HIV programmes. The current frameworks and measurement methods lack emphasis on effective coverage, occur infrequently, lack timeliness and limit the participation of KPs. The Effective Programme Coverage framework, which utilizes a programme science approach, provides an opportunity to assess gaps in various coverage domains and explore the underlying reasons for these gaps, in order to develop targeted solutions. We have demonstrated the application of this framework in partnership with the KP community in Nairobi, Kenya, using an expanded Polling Booth Survey (ePBS) method.

**Methods:**

Data were collected between April and May 2023 among female sex workers (FSWs) and men who have sex with men (MSM) using (a) PBS, (b) bio‐behavioural survey and (c) focus group discussions. Data collection and analysis involved both KP community and non‐community researchers. Descriptive analysis was performed, and proportions were used to assess the programme coverage gaps. The data were weighted to account for the sampling design and unequal selection probabilities. Thematic analysis was conducted on the qualitative data.

**Results:**

The condom programme for FSW and MSM had low availability (60.2% and 50.9%), contact (68.8% and 65.9%) and utilization (52.1% and 43.9%) coverages. The pre‐exposure prophylaxis (PrEP) programme had very low utilization coverage for FSW and MSM (4.4% and 2.8%), while antiretroviral therapy utilization coverage was higher (86.6% and 87.7%). Reasons for coverage gaps included a low peer educator‐to‐peer ratio, longer distance to the clinics, shortage of free condoms supplied by the government, experienced and anticipated side effects related to PrEP, and stigma and discrimination experienced in the facilities.

**Conclusions:**

The Effective Programme Coverage framework allows programmes to assess coverage gaps and develop solutions and a research agenda targeted at specific domains of coverage with large gaps. The ePBS method works well in collecting data to understand coverage gaps rapidly and allows for the engagement of the KP community.

## 1 INTRODUCTION

The recently launched UNAIDS Global AIDS Update 2023 highlights uneven progress in reducing new HIV acquisitions among key populations (KPs) [1]. It has now been established that scaling up programmes for KPs, maintaining high programme coverage and using real‐time data for continuous programme measurement and strategic adaptations is critical to ensure equitable access to health services for all [2]. Globally, though programmes for KPs have been scaled up in the last decade, measuring the coverage of essential services has been a challenge for national programmes [3]. Bio‐behavioural surveys (BBS) are commonly used and widely accepted tools for routinely measuring incidence and prevalence of HIV, behaviours and HIV programme coverage among KPs [4]. However, BBS are limited by their high financial and human resource intensity, particularly when conducted as frequently or in as many sites as needed to inform programmes with timely evidence and context [3, 5].

HIV prevention and treatment cascades have been globally adopted as useful frameworks for guiding programming with KPs [5]. Cascade analyses have been adapted in different contexts to measure and monitor programme coverage and outcomes using survey and programme data [6, 7]. Cascade analyses can highlight programmatic gaps in reaching and retaining KPs across the continuum of services and thereby identify areas for intensified work [8]. However, it has been challenging to adapt cascade analysis frameworks for HIV prevention due, in part, to the complexity of HIV prevention programmes and the availability of appropriate and timely data [9]. In recent years, there have also been discussions around examining HIV prevention and treatment programme performance through a service coverage lens to better understand health inequities and population‐level impact [10, 11, 12]. Effective Coverage is a useful construct to measure the proportion of a target population that receives a *health benefit* from an intervention or programme [13, 14]. Effective coverage unites individual and intervention characteristics into a single metric and offers a direct and flexible means to measure health system performance at multiple levels [15]. The concept of effective coverage can be applied in multiple public health programmes, and as such, the framework has been adapted and applied in several geographic and healthcare contexts [16, 17, 18].

**Figure 1:**
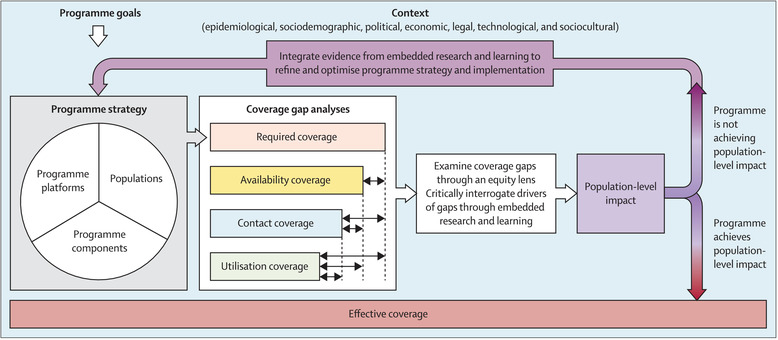
Effective Programme Coverage Framework. [57].

The HIV/sexually transmitted and blood‐borne infections (STBBI) Programme Science Initiative, a collective of leading public health experts with a joint interest in improving the quality, effectiveness and sustainability of HIV/STBBI programmes through a Programme Science approach [19, 20, 21] adapted the effective coverage framework (Figure 1) for HIV programmes. *The Effective Programme Coverage* framework [22] begins with understanding a programme's goal and strategy, which should typically consider the prioritized population, programme components or package of interventions/services offered by the programme, and platforms through which the interventions are delivered. A programme's interventions are monitored using a cascade that measures indicators for four dimensions of coverage—required‐, availability‐, contact‐ and utilization coverage as defined in Table S1. Coverage gap analyses are simultaneously performed by the programme team in partnership with the programme's end‐users through embedded research processes. To address gaps, evidence from gap analyses is fed back into the programme to inform adjustments to the programme strategy. The Effective Programme Coverage framework also emphasizes the importance for programmes to understand the ways in which context influences the programme's coverage cascade.

Using quantitative and qualitative data from an expanded Polling Booth Survey (ePBS) [23], this paper demonstrates how the framework (Figure 1) can be used to measure dimensions of programme coverage and understand factors contributing to gaps in coverage of HIV prevention programmes for female sex workers (FSWs) and men who have sex with men (MSM) in Nairobi County, Kenya. This paper uses the framework as a post‐hoc analysis tool to address the programme management and evaluation sphere of programme science [21].

## 2 METHODS

### 2.1 The Kenya key population programme

The Kenya KP programme was initiated in 2009 following the completion of the Modes of Transmission study [24], which found that FSW, MSM, prison populations and people who inject drugs (PWID) accounted for 33% of new HIV in Kenya. The first BBS was conducted during 2010−2011 and reported significantly higher HIV prevalence among FSW (29%), MSM (18%) and PWID (19%) [25], than the general population (5.6%) at that time [26]. As a part of the programme, mapping and population size estimation exercises have been conducted, providing good programme denominators. The most recent exercise (2020) estimated 197,000 FSW, 51,000 MSM, 35,000 PWID and 4300 transgender people in Kenya [27]. The KP programme, led by the Ministry of Health, has seen significant expansion over the last decade with high enrolment of estimated KPs into the programme: 73% for FSW, 82% for MSM, 71% for PWID through the needle syringe programme and 26% through opioid substitution therapy across more than 35/47 counties in Kenya [28]. More than 100 partners implement the programme [28], using a standard HIV combination prevention package as defined in the national guidelines [29].

### 2.2 Study site

The study site was Nairobi County, which has approximately 20% and 30% of the country's estimated FSW (*n* = 39,227) and MSM (*n* = 15,271) living or working in the County, respectively [27]. The County has identified several venue and non‐venue‐based sites where sex work and “cruising” takes place across its 17 sub‐counties [30]. Importantly, same‐sex sexual practices are criminalized across Kenya and all sex work was banned by Nairobi County in 2017 [31, 32]. This has contributed to high levels of violence, stigma and discrimination among FSW and MSM [33] in the County. Despite this, several implementing partners provide HIV prevention and treatment services to FSW and MSM, reaching almost 90% of the estimated population in the County [34]. This study was conducted by the University of Manitoba and Partners for Health and Development in Africa (PHDA), a local NGO, implementing the largest FSW and MSM programme in Nairobi, Kenya, in partnership with Nairobi County.

### 2.3 Study respondents

The study respondents included FSW and MSM. Eligible FSWs were those who identified as cisgender women, were aged 18 and above, and acknowledged having received money or gifts in exchange for sexual intercourse with a cisgender man at least once in the past 3 months. Eligible MSM were those who identified as cisgender men, were aged 18 and above, and reported at least one anal sex act with another cisgender man in the past 3 months. Additionally, potential respondents were required to: (a) be capable and willing to provide written or verbal informed consent; (b) self‐identify as an FSW or MSM; and (c) practice sex work or cruise within Nairobi County.

### 2.4 Data sources and data collection

This study employed both quantitative and qualitative data collection and analysis through an “ePBS” method, which expands upon the traditional PBS method [35] by incorporating biological and qualitative data collection to the traditional PBS. Quantitative methods included a PBS (discussed further below) and a brief, individualized BBS. Qualitative data were derived from focus group discussions (FGDs) conducted among a subset of PBS session respondents. Four study teams collected data simultaneously in four locations every day. Primary data were collected in the sampled sites during the evening, between 5 and 8 pm for 28 working days between April and May 2023. Data collection teams included five KP community members, four non‐community researchers, four clinicians, four HIV testing service (HTS) counsellors and two social scientists. The teams received 5 days of training, including pilot testing of the tool and the sampling method. Participation in the PBS and BBS components of the study took approximately 2 hours; participation in the FGD required an additional 30 minutes. Per protocol and ethics approval, all respondents were provided Ksh. 500 (approximately 3 USD) to cover their travel expenses and loss of time. Those who participated in the FGD were provided an additional Ksh. 500.

### 2.5 Polling booth survey

PBS is a group interview facilitated by a trained researcher, in which respondents are provided a private booth containing colour‐coded “yes,” “no” and “not applicable” ballot boxes and a set of numbered “voting” tokens corresponding to each PBS questionnaire item. The researcher reads each PBS question aloud and respondents provide their responses by placing the appropriately numbered token in the relevant ballot box. In this study, there were two separate PBS questionnaires for FSW and MSM respondents. The PBS method has been described in detail elsewhere [36, 37].

Two‐stage random sampling was adopted to recruit respondents. The first stage of the sampling procedure relied on a master list of physical sites where FSW and MSM met their sexual partners, previously established during a mapping and KP size estimation exercise conducted in 2018 [30]. The list of sites was then validated by the research team by conducting on‐site visits for observation and completing a validation form. This process took place during the preparatory stage of the study, and allowed for the inclusion of new sites that had emerged and the removal of those that had shut down. The team validated 1586 FSW sites and 235 MSM sites. The population size estimates for FSW and MSM in each of the validated sites were simultaneously confirmed using a standard validation tool. At the first stage of sampling, considering the sample size and planned PBS sessions, 64/1586 and 35/235 sites from the FSW and MSM site lists, respectively, were randomly selected after stratifying the sites by sub‐County and typology. Subsequently, each of these sites was randomly allocated a day of the week for data collection. At the second stage of sampling, respondents were randomly sampled from the selected sites on the allocated day of the week during evening hours (a time when higher numbers of FSW and MSM congregate). The respondents were taken to a safe space at the site or near the site where all the data were collected by two researchers: one KP community researcher and one non‐KP researcher. The community researcher focused on eligibility screening, mobilizing selected respondents to the study location, providing clarification on PBS questions and collating response cards for tallying.

### 2.6 Individual bio‐behavioural surveys

Following the completion of PBS sessions, those who provided informed consent were directed to the study clinical officer and the HTS counsellor for a face‐to‐face BBS. The HTS counsellor provided pre‐test counselling and conducted a rapid HIV test following national guidelines [38]. After that, each respondent met a clinical officer for a brief (18 questions for FSW and 21 questions for MSM) behavioural survey, followed by biological sample collection (blood and urine).

Among respondents receiving a positive HIV rapid test result, venous blood samples (5 ml) were collected and sent to the PHDA laboratory for HIV viral load testing, following national guidelines [39]. To maintain confidentiality, urine samples were collected from all respondents, but only samples from respondents reporting taking pre‐exposure prophylaxis (PrEP) during the behavioural survey were sent to the PHDA laboratory. Urine samples were tested for tenofovir using an assay developed and validated by the University of California, San Francisco in conjunction with Abbott Laboratories [40]. BBS data were collected by study teams within the same locations where PBS sessions had taken place.

### 2.7 Focus group discussions

FGDs were conducted with FSW and MSM to understand the challenges experienced while accessing and using HIV services in Nairobi County. For both KP groups, all respondents from every fifth PBS session (10−12 individuals) were invited to participate in FGD, following a separate informed consent procedure. An FGD guide, developed to probe for barriers that contributed to gaps in availability, contact and utilization coverage of HIV services, was used. All FGDs were facilitated by social scientists trained in qualitative methods and were conducted in the same sites as PBS sessions, immediately following the completion of the PBS. All discussions took place in Kiswahili, Sheng (a local slang common in urban Nairobi), or a combination thereof. All FGDs were audio‐recorded, translated and transcribed verbatim by the study team.

### 2.8 Data analysis

PBS and BBS data were captured in real time using SurveyCTO. Aggregate responses from each PBS session and individual data from BBS respondents were entered into separate data modules in SurveyCTO. Data were exported to SPSS 28.0 for analyses. Data were weighted, taking into account the sampling design, to adjust for unequal selection probabilities and confidence intervals (CI) were calculated. Descriptive analyses of PBS and BBS data were performed separately to address study objectives.

FGD data were simultaneously translated and transcribed into English by the qualitative study team. Qualitative notes taken during the FGD were incorporated into a final transcript to provide appropriate context and detail where necessary. Data were analysed using thematic analysis to systematically capture recurring concepts, ideas and topics that emerged during the discussions.

### 2.9 Measuring and defining programme coverage

#### 2.9.1 Condoms

In this study, we follow the targets set by the Kenya key population programme as documented in the national guidelines [29]. Hence, the required coverage indicator for condoms was set to 100%. Condom availability coverage is measured using the PBS question (Table S2): *During the past 1 month, was there a time when you intended to use a condom with any of your sexual partners but did not use it because a condom was not available at that time and place?* As government condoms are largely distributed for free by peer educators in Nairobi County, condom contact coverage is measured using the PBS question: *In the last 3 months, were you met by a peer educator from the programme?* Finally, condom utilization coverage is measured using the PBS question: *During the past 3 months, was there any occasion when you had sex with any paying client/sexual partner without using a condom?*

#### 2.9.2 Pre‐exposure prophylaxis

PrEP‐required coverage was set at 100% of all respondents who tested negative for HIV when tested during the study. The PrEP availability coverage is also considered at 100% as all clinics for KPs in Nairobi offer PrEP services. The PrEP contact coverage is measured using the BBS question (Table S2): *What HIV services have you received in the last one year?* According to Kenyan national guidelines, PrEP‐related counselling and assessment of behaviours that put one at risk of HIV acquisition initiates at the time of HIV testing. As such, all respondents who reported having an HIV test in the last year were considered to have had contact coverage for PrEP. Utilization coverage for PrEP was measured using the results of the rapid, urine‐based test for tenofovir.

#### 2.9.3 Antiretroviral therapy

Required coverage for antiretroviral therapy (ART) was set at 100% of all respondents who tested positive for HIV during the ePBS study, as per national guidelines [29]. All KP clinics in Nairobi provide ART services for KP who are living with HIV, so ART availability coverage was assumed to be 100%. ART contact coverage and ART utilization coverage was measured using the questions from BBS (Table S2): *Have you ever been on ART? and, Are you currently on ART?* respectively.

### 2.10 Ethics approval

The study received ethics approval from the Health Research Ethics Board at the University of Manitoba, Canada (HS25883) and the AMREF Ethics and Scientific Review Committee, Kenya (AMREF‐ESRC P1365/2022).

## 3 RESULTS

The study recruited a total of 759 FSW (99% response rate) and 398 MSM (95% response rate) respondents, across 64 and 35 PBS sessions, respectively (Table 1). In total, 758 FSW (one respondent declined to participate in the BBS) and 398 MSM participated in the BBS, and all provided blood and urine samples. Out of all the respondents, 171 respondents (101 FSW and 70 MSM) tested positive for HIV by rapid test. The rapid tenofovir urine test was conducted on 56 FSW samples and 17 MSM samples from respondents reporting currently using PrEP during BBS. A total of 20 FGD (14 with FSW and 6 with MSM) were conducted over the course of the study.

**Table 1:** Recruitment of respondents

	Female sex workers	Men who have sex with men
Total respondents	759	398
PBS respondents	759	398
BBS respondents	758	398
HIV rapid tests conducted	758	398
HIV‐positive results	101	70
Urine tests conducted (on those who reported using PrEP in BBS)	57	17

Abbreviations: BBS, Bio‐behavioural surveys; PBS, Polling Booth Survey; PrEP, pre‐exposure prophylaxis.

Table 2 presents the characteristics of study respondents, by KP group. Overall, a large proportion of FSW respondents (45%) were within the age range of 25−34 years, whereas most MSM respondents (54%) were below 25 years. The mean age for FSW and MSM respondents was 37.2 and 29.2 years, respectively. A large proportion of FSW respondents (51%) had been engaged in sex work for more than 10 years, with a mean duration of 11.4 years. The majority of MSM respondents (46%) were involved in same‐sex sexual activity for less than 5 years, with a mean duration of 6.4 years. Among the FSW respondents, 68% reported having more than 5 sex acts per week, with a mean of 11.7 acts per week. However, among the MSM respondents, 58% had 2 or fewer sex acts per week, with a mean of 3.1 acts per week.

**Table 2:** Socio‐demographic and sex work/same‐sex sexual activity‐related characteristics of female sex workers and men who have sex with men, Nairobi, Kenya, 2023

Characteristics	FSW (*n* = 758)	MSM (*n* = 398)
**Age**		
<25 years	17.8%	54.2%
25−34 years	45.4%	38%
35+ years	36.8%	7.9%
Mean age	37.2 (SD = 7.7)	29.2 (SD = 6.2)
Median age	32.0 (IQR = 12.0)	24.0 (IQR = 5.0)
**Duration in sex work/same‐sex sexual activity**		
<5 years	22.6	46.1
5−9 years	26.3	35.2
10+ years	51.1	18.7
Mean duration	11.4(SD −8.0)	6.4 (SD −5.1)
Median duration	16.0 (IQR = 14.0)	7.0 (IQR = 12.0)
**Number of sex acts per week **		
< = 2 sex acts	8.9	58.4
3−4 sex acts	23	26.3
5+ sex acts	68.1	15.3
Mean number of sex acts per week	11.7 (SD = 12.3)	3.1 (SD = 4.2)
Median number of sex acts per week	7 (IQR = 11.0)	2.0 (IQR = 2.0)

Abbreviations: FSW, female sex workers; IQR, interquartile range; MSM, menwho have sex with men; SD, standard deviation.

### 3.1 Programme coverage cascade—FSW

**Figure 2:**
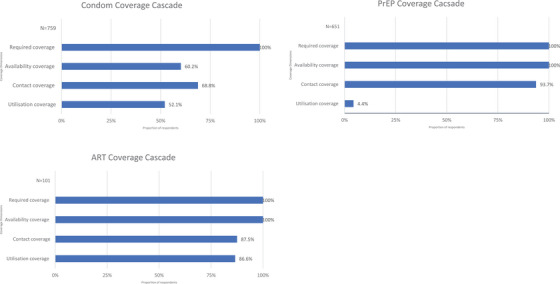
Programme Coverage Cascade for female sex workers.

Figure 2 (Tables S3−S5) presents the programme coverage cascade for FSW in Nairobi County. Condom availability coverage was 60.2% [95% CI: 56.7−63.7], contact coverage was 68.8% [95% CI: 65.5−72.1] and the utilization coverage was 52.1% [95% CI: 48.5−55.7] among respondents. For PrEP and ART, while the availability coverage was 100% for both, contact coverage was 93.7% [95% CI: 91.7−95.6] and 87.5% [95% CI: 81.1−93.9], respectively, and utilization coverage was 4.4% [95% CI: 2.8−6.0] and 86.6% [95% CI: 81.7−94.5], respectively.

#### 3.1.1 Programme coverage cascade—MSM

**Figure 3:**
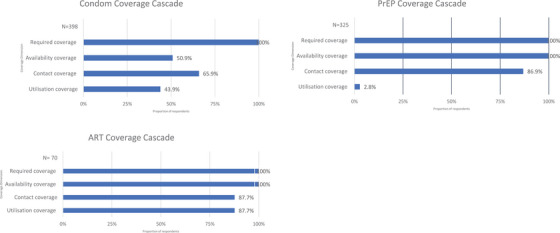
Programme Coverage Cascade for men who have sex with men.

Figure 3 (Tables S6−S8) shows the programme coverage cascade for MSM. Condom availability coverage was 50.9% [95% CI: 46.0−55.8], contact coverage was 65.9% [95% CI: 61.2−70.6] and utilization coverage was 43.9% [95% CI: 39.9−48.8] among respondents. For PrEP and ART, while availability coverage was 100% for both, contact coverage was 86.9% [95% CI: 83.0−90.7] and 87.7% [95% CI: 80.1−95.4], respectively, and utilization coverage was 2.8% [95% CI: 1.0−5.0] and 87.7% [95% CI: 85.5−98.1], respectively.

#### 3.1.2 Exploring and understanding identified gaps in prevention programme coverage

Through FGD, respondents shed light on strategies that supported high coverage of HIV services and highlighted several reasons for gaps in coverage.

#### 3.1.3 Strategies that support high contact and utilization coverage

FSW and MSM respondents mentioned peer educators as being predominantly responsible for providing essential services and supplies such as condoms, lubricants, health education and referral services. Respondents consistently described their experiences with peer educators as positive, attributing this to their shared identities, trustworthiness, dependability and willingness to go beyond the scope of their job to support the peers.

*I like the peer educators because they are really confidential, and they will keep your information private. So, when they give you the supplies like the condoms you will not hear it from anyone else*. (FSW, PBS 6)

Most FSW and MSM respondents also reported visiting facilities on a weekly, monthly and quarterly basis, or as needed. Although they might be registered with a specific clinic, they would also seek services from other clinics in the County if necessary.

“*I am a member of clinic A (name of clinic), and I joined in 2021. When I don't have money for transport, I visit the nearest facilities like organisation X (name of an implementing partner) for services and supplies. I go to the facility every three months or as per the appointment booked for me*.” (MSM, PBS 91)

Condoms emerged as a key prevention product, especially among FSW, who expressed a preference for the “triple benefits”—prevention of pregnancy, HIV and Sexually Transmitted Infections (STI)—provided by condoms. However, the FSW respondents also mentioned that when condoms are unavailable, then they may use PrEP.

*I feel that condoms are the best because they protect against getting unwanted pregnancies…STIs like syphilis, gonorrhoea…They also protect us against contracting HIV. When I don't get the condoms, I use…or I protect myself using PrEP. However, I also know that PrEP will not assist in preventing other diseases like STIs or even unwanted pregnancies*. (FSW, PBS 15)

#### 3.1.4 Reasons for low contact coverage

In terms of the reasons for gaps in contact coverage, the FSW and MSM respondents shared the need for more peer educators to ensure an optimal peer educator‐to‐peer ratio. The respondents also emphasized their desire for additional training for the peer educators to be better equipped to support and manage needs beyond HIV.

*Yeah, they (peer educators) are very okay, and I like their services. They just need to be trained a little bit more because you know I may be depressed, or I may be having some psychosocial issues, but you find that the peer educator cannot assist you at that point. They don't have the knowledge to handle such matters…* (MSM, PBS 68)

Distance and long travel times were commonly mentioned as barriers to regular contact with clinics. FSW respondents also cited the high cost of travel as a significant barrier to accessing clinical services.

*I think it's better to bring these services near us because going to a distant facility is also costly…You might not even have the money to pay fare. So, it is better if these services are brought near us so that one can effectively seek treatment or services*. (FSW, PBS 9)

Visiting a clinic that provides prevention and treatment services also facilitates KPs’ access to other services like visiting a clinic for HIV testing can support entry into the PrEP programme or access to condoms. However, the MSM respondents reported experiences of discrimination and stigma in the clinics and expressed a preference for clinics specifically meant for them rather than integrated services.

*So, my suggestion is that all the MSM clinics should be detached from this public place like we should have our own clinics not a clinic where the general public can access services. It should be a place where when you decide to visit you don't go like trying to hide yourself*. (MSM, PBS 70)

“*Okay in the clinics we should be divided like MSMs to have their own facility and FSWs to have their own facilities. We don't want a scenario where someone is maybe a cross‐dresser, and they feel so uncomfortable getting services together with the female sex workers. They should just be divided so that there is more privacy*.” (MSM, PBS 68)

#### 3.1.5 Reasons for utilization coverage gaps

Within the clinics, long waiting times, unsupportive staff and non‐availability of services during the weekend, holidays or late evenings were cited as barriers to the utilization coverage by FSWs.

*Again, for the facilities, they should increase the working hours because sometimes you might go there, you have risked with a client, and you reach a facility and find they have already closed*. (FSW, PBS 50)

All respondents also expressed concern about experiencing shortages of condoms and lubricants, which contribute to gaps in utilization. Discussions indicated that the respondents did not receive free government condoms regularly in the last year. They also reported that even if condoms were broadly available, they were not always available in all sites.

*There are those who face challenges getting these products and services. You find that one might be in need of the products or the services but the ways of getting them is hard. So, like they go to a health facility and are told that the services or products are not available*. (FSW, PBS 25)

*“I've faced situations where condoms are not available in all settings. For example, in some public places like clubs or parties, they don't provide condoms. So, if you don't have your own, you are left with no option but to engage in risky behaviour*.” (MSM, PBS 68)

While PrEP is available, utilization was low, and some of the reasons were related to the product itself and its branding. FSW and MSM shared that PrEP medication had undesirable side effects, which led to medication adherence issues. Additionally, respondents mentioned that the PrEP drugs were branded and packaged in the same way as ART, which created a barrier to using PrEP due to anticipated stigma. The respondents recommended that the KPs need more awareness about PrEP.

“*A person like me, I use condoms more because things like PrEP and PEP look like ARVs, so when someone sees you using them, they might think you have HIV, and you will experience some discrimination*.” (MSM, PBS 80)

“*For me it is on the PrEP drug, a lot of awareness needs to be created when it comes to PrEP. Most people are not aware of the PrEP and they have a lot of theories when it comes to PrEP*.” (FSW, PBS 40)

## 4 DISCUSSION

Our study in Nairobi, Kenya shows that there are large coverage gaps in condom programming for FSW and MSM in terms of availability‐, contact‐ and utilization coverage. The FGD respondents explained that the low contact coverage with peer educators is largely due to the insufficient number of peer educators available through the KP programmes. Peer education has been an integral part of the KP programme in Kenya. Peer educators provide life‐saving commodities and information including condoms, lubricants, health education and referrals for clinical services [41]. In Kenya, micro‐planning with adequate peer‐educator ratios has been found to be an effective approach to scaling up HIV prevention programmes among KPs, resulting in high levels of programme uptake and service utilization [42]. FSW and MSM community members reported that the shortage of free condoms provided by the government is a reason for the low utilization coverage of condoms. This underscores the concerns regarding condom supply shortage raised by activists and civil society organizations in Kenya since 2022 [43, 44]. FSWs have expressed a strong preference for using condoms as a primary HIV prevention strategy, as they also provide protection against other sexually transmitted infections and pregnancy.

In relation to the PrEP programme, there are high gaps in utilization coverage among all respondents. Though PrEP availability coverage is higher than condom availability coverage, PrEP utilization coverage is much lower than condoms. In the quantitative and qualitative assessment, respondents reported frequent visits to clinics, especially for HIV testing. There may have been missed opportunities to counsel the KPs on PrEP and facilitate its use after HIV testing. Through qualitative inquiry, the respondents stated that lack of knowledge, stigmatizing attitudes of healthcare providers, experienced and anticipated side effects, and branding of PrEP drugs are important barriers to use. These findings are consistent with earlier studies that identified low PrEP‐related knowledge, specific concerns about side effects, being viewed as living with HIV if taking PrEP and stigma by healthcare providers within service delivery settings as barriers to access and use of PrEP among male and female sex workers in Kenya [45, 46]. Other studies from similar context also noted daily pill taking, and low perceived risk were some of the reasons given by KPs for discontinuing PrEP [45, 47, 48].

Our study also shows that in the treatment programme, the contact coverage and utilization coverage are higher than in the condom or PrEP programmes. However, the provision of ART for FSW and MSM living with HIV in Nairobi is still below the UNAIDS target of 95% [49]. Though respondents in our study did not share the barriers to ART enrolment or utilization, other studies have shown that low knowledge, negative perceptions about ART, poor pill‐taking practices, inadequate counselling and preparation of the client and experience or fear of stigma and prejudice by healthcare workers are associated with low ART utilization and low viral suppression [50, 51].

A strong, flexible monitoring framework is the starting point for identifying gaps and promoting actions aimed at improving the coverage of HIV programmes for KPs [52, 53]. PBS is a well‐established method known for its ability to elicit higher reporting of behaviours related to the acquisition of HIV among KPs due to the improved anonymity and confidentiality of its secret ballot voting [54]. The Kenya KP programme has found PBS to be a very reliable methodology for collecting data within a short period of time at a low cost, engaging KPs in data collection and analysis and suitable for monitoring programme outcomes on a regular basis to guide the continuous refinement of the programme [37].

In our study, we expanded the existing PBS method to include key individualized data, biological samples and qualitative inquiry to address some of the identified limitations related to PBS earlier [54]. A particular strength of the novel ePBS method lies in its two‐stage random sampling approach, which aims to generate a representative sample of KP community members, regardless of whether they have been reached by or connected with prevention programmes. Importantly, the meaningful involvement of the affected community in monitoring programmes improves the value of these activities [55]. The ePBS method offers these opportunities as the simplicity of the tool encourages KPs to play critical leadership roles in study design, implementation and analysis. It is important to note that careful consideration must be given to the risks and potential safety issues that could arise during data collection among KPs, as particular sex practices and sex work are stigmatized, or even criminalized, in many contexts [52]. Additionally, it should be noted that individual KPs do not necessarily share a similar risk of HIV acquisition, hence the ePBS questions in the future should be designed to capture the heterogeneity in the population and measure outcomes of combination prevention as stated in the Global AIDS Strategy 2021−2026 [56]. These targets consider the interactions between different prevention methods, including condoms, PrEP, U = U, which together, should contribute to the achievement of the targets [12].

Further areas of research emerging from the analysis and consultations are (a) understanding the variability in coverage gaps based on geography (sub‐County or sites), age or entry into sex work; (b) practical strategies to address stigma and discrimination, specifically in integrated public health facilities; and (c) understanding the influence of the context on different domains of programme coverage.

Here, we suggest that, together, the Effective Programme Coverage framework and the ePBS method are effective tools to monitor, assess and understand programme coverage and gaps. Although there are several cascade frameworks [5] that can be used to assess programme gaps, the use of the effective programme coverage framework allows programmes to specifically examine one critical dimension of the programme, that is coverage. Exploring gaps in different dimensions of coverage (required, availability, contact and utilization) allows identification of gaps in a specific domain and finding targeted solutions and research questions to address the gaps in the domain.

### 4.1 Limitations

The study has several limitations. Firstly, the study was not originally conceptualized and designed using the Effective Programme Coverage framework. Hence, our analyses to generate estimates for the coverage cascade were imperfect. In particular, the PBS tool lacked questions directly related to availability coverage. For future rounds of ePBS, it will be feasible to more effectively quantify and understand coverage gaps by designing the tools to align more accurately with the coverage domains outlined in the framework. Secondly, the study sampled respondents from the physical sites only, missing out the FSW and MSM who practice in the virtual sites. In the future, sampling for such studies should consider all sites. Additionally, though stigma and discrimination were expressed as barriers to contact and utilization coverage, probing around these structural factors was not explicitly included in the qualitative inquiry. In the future, it would be prudent for ePBS studies in Kenya to intentionally collect relevant data to create a coverage cascade for mental health, violence and stigma prevention and response programmes, similar to condom, PrEP and ART.

## 5 CONCLUSIONS

The Effective Programme Coverage framework provides guidance for HIV prevention programmes for KPs to measure programme coverage gaps and identify missed opportunities and reasons for the coverage gaps and inequities for the programme to address in a timely manner. Employing the ePBS method allows for the collection of sensitive behavioural data in a rapid way while maintaining confidentiality and facilitating meaningful community involvement and leadership in the data collection and analysis processes, with the goal to address gaps in programme coverage in real time.

## COMPETING INTERESTS

The authors declare that they have no competing interests.

## AUTHORS’ CONTRIBUTIONS

PB, LM, MLB, JB and GG conceptualized the paper. PB wrote the first draft of the paper and SI, LM, FE, GG and MLB contributed in writing different sections of the paper and reviewing drafts. PB, JK, RK, AK and HM generated the data and managed the data collection process. SI supported the training for data collection, data cleaning and data analysis. CO and BKK supported the data collection and analysis of the qualitative research. SY‐NS, SM, PM and MG reviewed the manuscript and provided feedback. All co‐authors contributed to the design of the study, reviewed the paper and made revisions.

## FUNDING

This study is made possible by the support of the Bill & Melinda Gates Foundation (BMGF) under grant ID INV 50927.

## DISCLAIMER

The views expressed herein are those of the authors and do not necessarily reflect the official policy or position of BMGF.

## Supporting information


**Table S1**: List of definitions

**Table S2**: List of questions

**Table S3**: Condom coverage cascade for FSW in Nairobi, Kenya, April−May 2023

**Table S4**: PrEP coverage cascade for FSW in Nairobi, Kenya, April−May 2023

**Table S5**: ART coverage cascade for FSW living with HIV in Nairobi, Kenya, April−May 2023

**Table S6**: Condom coverage cascade for MSM in Nairobi, Kenya, April−May 2023

**Table S7**: PrEP coverage cascade for MSM in Nairobi, Kenya, April−May 2023

**Table S8**: ART coverage cascade for MSM living with HIV in Nairobi, Kenya, April−May 2023

## Data Availability

Data from this study are available to researchers who meet the criteria for access to confidential data through the study data manager Tony Kariri, available at Tkariri@csrtkenya.org. The investigators are ethically bound to safeguard study materials, including data. Data usage by anyone other than the listed co‐investigators of the study requires permission from the AMREF and UoM ethics committee. The BBS and FGD data include sensitive data. As sex work and same‐sex relationships are criminalized in Kenya and considering the prevailing antagonistic environment against LGBTQI+ populations, we think it would be unsafe to publicly share the data.
