# Supplementary material for: Applying the Effective Programme Coverage framework to assess gaps in HIV prevention programmes for female sex workers and men who have sex with men in Nairobi, Kenya: findings from an expanded Polling Booth Survey
Source: J Int AIDS Soc. 2024 Jul 10;27(Suppl 2):e26240. doi: 10.1002/jia2.26240 (PMC11233849; doi:10.1002/jia2.26240)
Supplement: Supplementary file 1 — Table S1: List of definitions [file JIA2-27-e26240-s002.docx]

**Table S1.** Coverage cascade steps definitions, as defined in the Effective Programme Coverage framework^[[1]](#endnote-1)^

| Coverage cascade step | Definition |
| --- | --- |
| Required coverage | - Defines the estimated key populations that will benefit most from specific programme services, within a given context. - Establishes the target for programme outputs and outcomes that are monitored during implementation - Typically acts as denominator for subsequent cascade steps |
| Availability coverage | - Measures programme’s capacity to provide specific programme services to key populations who require programme coverage - Proportion of the specific programme services available to key populations within prioritised geographies to meet required coverage targets - Availability coverage targets set separately for different programme services (condom distribution, provision of PrEP or ART) for different sub populations and geographies |
| Contact coverage | - Measures contact between the programmes providing specific services and key populations relative to the required coverage targets |
| Utilization coverage | - Measures uptake of specific programme services by key populations in relation to the established required coverage target - Compared to contact coverage, utilization coverage is more directly related to observed changes in monitored programme outcomes. |

1. McClarty, L.M., Becker, M.L., García, P.J., Garnett, G.P., Dallabetta, G.A., Ward, H., Aral, S.O. and Blanchard, J.F., 2023. Programme science: a route to effective coverage and population-level impact for HIV and sexually transmitted infection prevention. *The Lancet HIV*, *10*(12), pp.e825-e834. [↑](#endnote-ref-1)
