# Supplementary material for: Applying the Effective Programme Coverage framework to assess gaps in HIV prevention programmes for female sex workers and men who have sex with men in Nairobi, Kenya: findings from an expanded Polling Booth Survey
Source: J Int AIDS Soc. 2024 Jul 10;27(Suppl 2):e26240. doi: 10.1002/jia2.26240 (PMC11233849; doi:10.1002/jia2.26240)
Supplement: Supplementary file 2 — Table S2: List of questions [file JIA2-27-e26240-s008.docx]

Table S2. List of questions

| Variable | Questions |
| --- | --- |
| **Figure 2** | |
| Condom Availability Coverage | PBS FSW Q05, During the past 1 month, was there a time when you intended to use a condom with any of your sexual partners but did not use it because a condom was not available at that time and place? |
| Condom Contact Coverage | PBS FSW Q21, In the last 3 months, were you met by a peer educator from the program? |
| Condom Utilisation Coverage | PBS question, Complement of Q2, During the past 3 month, was there any occasion when you had sex with any paying client without using a condom? |
| PrEP Availability Coverage | All KP clinics that offer PrEP in Nairobi |
| PrEP Contact Coverage | FSW Individual questionnaire Q13, What HIV services have you received in the last one year (Those who reported HIV testing as a service) |
| PrEP Utilisation Coverage | FSW Individual questionnaire Q18, Those FSW who tested positive for tenofovir based on rapid urine test |
| ART Availability Coverage | All KP clinics that offer ART in Nairobi |
| ART Contact Coverage | FSW Individual questionnaire Q8, Have you ever been on ART? |
| ART Utilisation Coverage | FSW Individual questionnaire Q9, Are you currently on ART? |
| **Figure 3** | |
| Condom Availability Coverage | PBS MSM Q13, During the past 1 month, was there a time when you intended to use a condom with any of your sexual partners but did not use it because a condom was not available at that time and place? |
| Condom Contact Coverage | PBS MSM Q30, In the last 3 months, were you met by a peer educator from the program? |
| Condom Utilisation Coverage | PBS MSM question, complement of Q12, During the past 3 month, was there any occasion when you had sex with any sexual partners without using a condom? |
| PrEP Availability Coverage | All KP clinics that offer PrEP in Nairobi |
| PrEP Contact Coverage | MSM Individual questionnaire Q16, What HIV services have you received in the last one year (Those who reported HIV testing as a service) |
| PrEP Utilisation Coverage | MSM Individual questionnaire Q21, Those MSM who tested positive for tenofovir based on rapid urine test |
| ART Availability Coverage | All KP clinics that offer ART in Nairobi |
| ART Contact Coverage | MSM Individual questionnaire Q11, Have you ever been on ART? |
| ART Utilisation Coverage | MSM Individual questionnaire Question 12, Are you currently on ART? |
