# Supplementary material for: Applying the Effective Programme Coverage framework to assess gaps in HIV prevention programmes for female sex workers and men who have sex with men in Nairobi, Kenya: findings from an expanded Polling Booth Survey
Source: J Int AIDS Soc. 2024 Jul 10;27(Suppl 2):e26240. doi: 10.1002/jia2.26240 (PMC11233849; doi:10.1002/jia2.26240)
Supplement: Supplementary file 3 — Table S3: Condom coverage cascade for FSW in Nairobi, Kenya, April−May 2023 [file JIA2-27-e26240-s003.docx]

**Table S3. Condom coverage cascade for FSW in Nairobi, Kenya, April – May, 2023**

|  | Unweighted n | Weighted  % [95% CI] |
| --- | --- | --- |
| FSW who require condoms^#^ - Required Coverage (N=759) | 759 | 100 |
| FSW who reported finding condoms when needed – Availability Coverage (N=754) * | 456 | 60.2 [56.7-63.7] |
| FSW who reported being in contact with peers - Contact coverage (N=748) ** | 511 | 68.8 [65.5-72.1] |
| FSW who reported using condoms consistently – Utilisation coverage (N=751) *** | 385 | 52.1 [48.5-55.7] |

Data Source: Polling Booth Survey. Survey questions are detailed in S1

FSW: Female sex works

^#^It is estimated that all FSW will require condoms

*5 FSW respondents did not respond to this question

**11 FSW respondents did not respond to this question

***8 FSW respondents did not respond to the question
