# Supplementary material for: Applying the Effective Programme Coverage framework to assess gaps in HIV prevention programmes for female sex workers and men who have sex with men in Nairobi, Kenya: findings from an expanded Polling Booth Survey
Source: J Int AIDS Soc. 2024 Jul 10;27(Suppl 2):e26240. doi: 10.1002/jia2.26240 (PMC11233849; doi:10.1002/jia2.26240)
Supplement: Supplementary file 4 — Table S4: PrEP coverage cascade for FSW in Nairobi, Kenya, April−May 2023 [file JIA2-27-e26240-s001.docx]

**Table S4. PrEP coverage cascade for FSW in Nairobi, Kenya, April – May, 2023**

|  | Unweighted n | Weighted  % [95% CI] |
| --- | --- | --- |
| FSW who require PrEP^#^- Required Coverage (N= 651) | 651 | 100 |
| FSW who reported visiting the clinic for HIV testing - Contact coverage (N=637) * | 599 | 93.7 [91.7-95.6] |
| FSW who tested positive for tenofovir – Utilisation coverage (N=651) ** | 27 | 4.4 [2.8-6.0] |

Data Source: Behavioural and biological survey. Survey questions are detailed in S1

FSW: Female sex works

PrEP: Pre Exposure Prophylaxis

# It is estimated that all FSW respondents who tested negative for HIV will require PrEP

*14 FSW respondents did not respond to this question

** Urine samples of only those 56 FSW respondents who reported using PrEP in the survey were tested for tenofovir.
