# Supplementary material for: Applying the Effective Programme Coverage framework to assess gaps in HIV prevention programmes for female sex workers and men who have sex with men in Nairobi, Kenya: findings from an expanded Polling Booth Survey
Source: J Int AIDS Soc. 2024 Jul 10;27(Suppl 2):e26240. doi: 10.1002/jia2.26240 (PMC11233849; doi:10.1002/jia2.26240)
Supplement: Supplementary file 5 — Table S5: ART coverage cascade for FSW living with HIV in Nairobi, Kenya, April−May 2023 [file JIA2-27-e26240-s007.docx]

**Table S5. ART coverage cascade for FSW living with HIV in Nairobi, Kenya, April – May, 2023**

|  | Unweighted n | Weighted  % [95% CI] |
| --- | --- | --- |
| FSW who require ART^#^- Required Coverage (N= 101) | 101 | 100 |
| FSW who reported ever taking ART - Contact coverage (N=101) | 90 | 87.5 [81.1-93.9] |
| FSW who reported currently taking ART – Utilisation coverage (N=101) | 89 | 86.6 [81.7-94.5] |

Data Source: Behavioural and biological survey. Survey questions are detailed in S1

FSW: Female sex works

ART: Antiretroviral Therapy

# It is estimated that all FSW respondents who tested positive for HIV will require ART
