# Supplementary material for: Applying the Effective Programme Coverage framework to assess gaps in HIV prevention programmes for female sex workers and men who have sex with men in Nairobi, Kenya: findings from an expanded Polling Booth Survey
Source: J Int AIDS Soc. 2024 Jul 10;27(Suppl 2):e26240. doi: 10.1002/jia2.26240 (PMC11233849; doi:10.1002/jia2.26240)
Supplement: Supplementary file 6 — Table S6: Condom coverage cascade for MSM in Nairobi, Kenya, April−May 2023 [file JIA2-27-e26240-s004.docx]

**Table S6. Condom coverage cascade for MSM in Nairobi, Kenya, April – May, 2023**

|  | Unweighted n | Weighted  % [95% CI] |
| --- | --- | --- |
| MSM who require condoms^#^ - Required Coverage (N=398) | 398 | 100 |
| MSM who reported finding condoms when needed – Availability Coverage (N=398) | 206 | 50.9 [46.0-55.8] |
| MSM who reported being in contact with peers - Contact coverage (N=398) | 272 | 65.9 [61.2-70.6] |
| MSM who reported using condoms consistently – Utilisation coverage (N=398) | 178 | 43.9 [39.0-48.8] |

Data Source: Polling Booth Survey. Survey questions are detailed in S1

MSM: men who have sex with men

^#^It is estimated that all MSM will require condoms
