# Supplementary material for: Applying the Effective Programme Coverage framework to assess gaps in HIV prevention programmes for female sex workers and men who have sex with men in Nairobi, Kenya: findings from an expanded Polling Booth Survey
Source: J Int AIDS Soc. 2024 Jul 10;27(Suppl 2):e26240. doi: 10.1002/jia2.26240 (PMC11233849; doi:10.1002/jia2.26240)
Supplement: Supplementary file 7 — Table S7: PrEP coverage cascade for MSM in Nairobi, Kenya, April−May 2023 [file JIA2-27-e26240-s005.docx]

**Table S7. PrEP coverage cascade for MSM in Nairobi, Kenya, April – May, 2023**

|  | Unweighted n | Weighted  % [95% CI] |
| --- | --- | --- |
| MSM who require PrEP^#^- Required Coverage (N= 325) | 325 | 100 |
| MSM who reported receiving HIV testing services - Contact coverage (N=297) * | 258 | 86.9 [83.0-90.7] |
| MSM who tested positive for tenofovir – Utilisation coverage (N=325) ** | 10 | 2.8 [1.0-5.0] |

Data Source: Behavioural and biological survey. Survey questions are detailed in S1

MSM: Men who have sex with men

PrEP: Pre-Exposure Prophylaxis

# It is estimated that all MSM who tested HIV negative will require PrEP

*28 MSM respondents did not respond to this question

** Urine samples of only 17 MSM respondents who reported using PrEP in the survey were tested for tenofovir
