# Supplementary material for: Applying the Effective Programme Coverage framework to assess gaps in HIV prevention programmes for female sex workers and men who have sex with men in Nairobi, Kenya: findings from an expanded Polling Booth Survey
Source: J Int AIDS Soc. 2024 Jul 10;27(Suppl 2):e26240. doi: 10.1002/jia2.26240 (PMC11233849; doi:10.1002/jia2.26240)
Supplement: Supplementary file 8 — Table S8: ART coverage cascade for MSM living with HIV in Nairobi, Kenya, April−May 2023 [file JIA2-27-e26240-s006.docx]

**Table S8. ART coverage cascade for MSM living with HIV in Nairobi, Kenya, April – May, 2023**

|  | Unweighted n | Weighted  % [95% CI] |
| --- | --- | --- |
| MSM who require ART^#^- Required Coverage (N= 70) | 70 | 100 |
| MSM who reported ever taking ART - Contact coverage (N=70) | 63 | 87.7 [80.1-95.4] |
| MSM who reported currently taking ART – Utilisation coverage (N=70) | 63 | 87.7 [84.5-98.1] |

Data Source: Behavioural and biological survey. Survey questions are detailed in S1

MSM: Men who have sex with men

ART: Antiretroviral Therapy

# It is estimated that all MSM respondents who were tested positive for HIV will require ART
